# Explainable AI for sentiment analysis of human metapneumovirus (HMPV) using XLNet

**DOI:** 10.1371/journal.pone.0351265

**Published:** 2026-09-21

**Authors:** Md Shahriar Hossain Apu, Md Saiful Islam, Tanjim Taharat Aurpa, Sharad Hasan

**Affiliations:** 1 Department of IoT and Robotics Engineering, University of Frontier Technology, Bangladesh, Bangladesh; 2 Department of Educational Technology and Engineering, University of Frontier Technology, Bangladesh, Bangladesh; 3 Department of Data Science and Engineering, University of Frontier Technology, Bangladesh, Bangladesh; Children’s National Hospital, George Washington University, UNITED STATES OF AMERICA

## Abstract

The outbreak of Human Metapneumovirus (HMPV) in China, which later spread to the UK and other countries, raised significant public concern due to its potential impact on vulnerable populations. While HMPV typically causes mild symptoms, its effects on the elderly and immunocompromised individuals prompted health authorities to emphasize preventive measures. Moreover, continuous monitoring of respiratory viruses like HMPV remains important, as new factors (such as emerging variants) could alter their behavior over time. These factors have led to mixed public reactions, with some individuals expressing anxiety while others exhibit carelessness regarding the virus. This paper explores how sentiment analysis can enhance our understanding of public reactions to HMPV by analyzing data from social media platforms like YouTube. It highlights the importance of tracking public sentiment—ranging from fear to trust—to guide health messaging, inform policies, address misinformation, and encourage compliance with preventive measures during outbreaks. This study focuses on the use of sentiment analysis to understand public reactions to HMPV during the 2024 outbreak. The research applies advanced transformer models, particularly XLNet, achieving an accuracy of 93.50% in sentiment classification tasks. Additionally, We incorporate explainable AI (XAI) through SHAP to provide transparency in how the model identifies key factors influencing public sentiment.

## 1 Introduction

Human Metapneumovirus (HMPV) is a respiratory virus that causes cold-like symptoms such as cough, sore throat, runny nose and wheezing. While most infections remain mild, severe cases can lead to pneumonia or exacerbate preexisting chronic conditions, including asthma and chronic obstructive pulmonary disease (COPD). Human metapneumovirus (HMPV) is an important respiratory virus that commonly causes acute infections, especially in children, older adults, and people with weakened immune systems [1]. The most vulnerable populations are high-risk groups, including young children under five, elderly individuals over 65 and immunocompromised patients. Infection is caused mainly by respiratory droplets and contaminated surfaces; most cases resolve spontaneously. However, critical cases may require medical intervention [2].

HMPV infections have a seasonal pattern, peaking in late winter and early spring [3]. This trend is attributed to the virus’s enhanced survival in colder conditions and increased human contact in indoor settings [4]. Recently, from the late 2024 there was a remarkable increase seen in the HMPV infection in the Northern Hemisphere, mainly in the countries like China, the United States of America and the United Kingdom. However, this increase has not caused a disruption in public health systems, though WHO is very closely monitoring the global situation [5].

Given the emerging concern around HMPV and its increasing global prevalence, there is a lack of comprehensive research focused on public sentiment regarding the virus. Traditional data sources for sentiment analysis, such as surveys or medical reports, are limited, especially in capturing real-time public discourse. To bridge this gap, we turn to YouTube, a popular platform where users often discuss personal experiences and concerns. YouTube comments provide a unique, real-time window into public sentiment and are rich with diverse, spontaneous reactions to health-related topics. As this is the first research of its kind on HMPV-related sentiment analysis, we chose to collect comments specifically related to HMPV in order to explore and understand the public’s feelings and reactions.

Healthcare organizations require knowledge of HMPV-related public sentiment because respiratory illnesses increase in frequency during winter months. Electronic sentiment tracking allows health organizations to uncover major public concerns while tackling misleading information for better outreach practices. Within natural language processing (NLP) one of its key research areas refers to sentiment analysis which classifies texts based on positive negative and neutral sentiment categories [6], involves classifying text into sentiment categories such as positive, negative or neutral. Traditional sentiment analysis methods, including rule-based and statistical models, often struggle with capturing contextual and nuanced meanings in text [7].

Transformer-based models like BERT, XLNet and RoBERTa provide much improved context understanding and performance in NLP tasks. The models exhibit great effectiveness when used for sentiment analysis tasks thanks to their superior ability to interpret text data complexity. The implementation of SHAP (Shapley Additive Explanations) as a XAI technique helps improve sentiment classification transparency by providing model decision interpretations and visualizations.

XLNet provides excellent performance in NLP tasks because it functions as an advanced autoregressive transformer approach that handles three essential tasks: Named Entity Recognition [8], Sentiment Analysis [9], and Emotion Detection [10]. The developers made XLNet as an advanced version of Transformer-XL to deal with the sequence length constraints of standard transformers. XLNet incorporates both autoregressive and autoencoding language models to extract multi-directional contexts through an efficient combined framework design that avoids their individual weaknesses. The system contains a Two-Stream Self-Attention attention mechanism alongside positional encoding as part of its architectural design. During pretraining XLNet deploys Permutation Language Modeling (PLM) as an alternative to BERT which uses masked language models. Through its unique approach XLNet surpasses BERT by winning competitions in 20 NLP benchmarks for downstream application tasks.

The study intends to build a sentiment analysis framework for HMPV-related discourse through the combination of transformer-based architectures and XAI techniques. The processed user comment database with annotations enables this system to achieve accurate sentiment detection through interpretable predictive explanations.

The main contributions of this study are as follows:

- Development of a robust sentiment analysis pipeline for HMPV-related text using state-of-the-art NLP techniques.
- Integration of SHAP to enhance the transparency and interpretability of sentiment classification models.
- Insights into public sentiment on HMPV that may support health communication and future research on online public discourse.

By integrating cutting-edge machine learning models with explainability, this study bridges the gap between computational advancements and their real-world applications in public health. The findings of this research can facilitate more effective and informed decision-making in response to HMPV outbreaks.

The remainder of this paper is organized as follows: the Literature Review section discusses relevant studies on sentiment analysis, HMPV-related research, and transformer-based models. The Methodology section describes the data collection and preprocessing procedures, sentiment labeling, model development, evaluation, and integration of Explainable AI (XAI) techniques. The Results section presents the performance of the evaluated transformer models and the interpretability analysis. The Discussion section interprets the findings, discusses their implications and limitations, and outlines the scope of public health inference. Finally, the Conclusion and Future Work section summarizes the key findings and presents directions for future research.

## 2 Literature review

Researchers discovered Human Metapneumovirus (hMPV) in 2001 after establishing it as the main respiratory infection responsible for affecting young children and elderly people and immune-compromised patients [11]. This virus is responsible for 5–10% of pediatric hospitalizations due to acute respiratory infections and can lead to severe conditions like bronchiolitis and pneumonia. The symptoms of hMPV infection often resemble those of respiratory syncytial virus and RT-PCR is the preferred diagnostic method due to the virus’s slow growth in cell culture. Although vaccine candidates are under development, no vaccines are currently commercially available and research continues to improve the understanding and treatment strategies for hMPV.

A 68-year-old immunocompetent male experienced severe pneumonia due to hMPV according to [12]. Hospitalization became necessary for the patient despite lack of major concurrent illnesses because his respiratory condition deteriorated. The testing through multiplex RT-PCR confirmed the diagnosis while medical scans showed the presence of viral pneumonia. The patient’s full recovery with supportive care proved the value of molecular diagnostics in proper diagnosis [13] and confirmed the potential benefits of future vaccines including IVX-A12.

Kannappan et al. [14] examine the growing significance of sentiment analysis in the digital landscape. The research sheds light on its function to develop websites and build social media profiles as well as operate digital platforms. Using sentiment analysis allows businesses to handle customer support needs while analyzing product reviews to protect their reputation through positive reviews. The analysis investigates Natural Language Processing (NLP) and Machine Learning (ML) collaboration where NLP tools interpret human language and Python enhances the effectiveness of sentiment analysis. Kavitha et al. [15] explore the application of sentiment analysis on social media data using NLP and ML techniques. The research demonstrates the significance of sentiment analysis from user-created content via ML tools Random Forest and Logistic Regression that achieve effective processing and interpretation. The research shows that NLP and ML methods can produce valuable conclusions from social media information to boost decision-making effectiveness. Online and social media platforms employ automated recognition methods to presume user preferences, sensitive attributes such as race, gender, sexual orientation, and opinions [16].

Srivastava et al. [17] review various approaches to sentiment analysis within NLP, focusing on techniques that detect the emotional tone of text. This study demonstrates how sentiment analysis extracts meaningful information from the rapidly expanding online content which includes text and images and audio and video materials. The study examines three standard NLP methods including Naïve Bayes and Support Vector Machines (SVM) in addition to the lexicon-based approach while identifying sentiment analysis challenges for upcoming developments which will provide tools to smaller businesses and the general public.

Jim et al. [6] present a comprehensive review of recent advancements and challenges in sentiment analysis, a critical area within NLP. Research evaluations demonstrate how this technology classifies text-based information into three categories to help businesses obtain critical data about customer emotions. This study investigates sentiment analysis through exploration of multiple sectors alongside processing methods including datasets and evaluation methods which support the analysis. Furthermore it explores how Machine Learning and Deep Learning and Large Language Models contribute to sentiment analysis. The authors suggest research plans for upcoming years to resolve the problems discovered in present-day analytical systems.

Gunasekaran [18] reviews various sentiment analysis techniques within NLP, including lexicon-based, machine learning, deep learning, and hybrid approaches. The study demonstrates why sentiment analysis stands essential when processing customer feedback while helping marketing strategies and political processes. An analysis of Twitter serves as the main example to explore sector applications and assessment techniques which aid in advancing sentiment analysis efficiency and accuracy through dataset and metric comparisons.

Chong et al. [19] investigates NLP techniques in tweet sentiment analysis through subjectivity classification and semantic association and polarity classification steps. Better results than typical sentiment tools become achievable because the tool uses sentiment lexicons together with grammatical relationships. Specific natural language processing techniques are necessary to analyze sentiments within social media environments according to the research findings.

Jain et al. [20] focus on real-time sentiment analysis using multimedia inputs such as audio, video and text to interpret emotions. They compare techniques like Support Vector Machines (SVMs), Bayesian Networks, and Convolutional Neural Networks (CNNs) for sentiment classification. Their research leads to the development of a real-time sentiment analysis system to help users assess daily attitudes and receive relevant recommendations.

Wankhade et al. [21] provide a comprehensive survey on sentiment analysis, reviewing methods, applications, and challenges. This analysis demonstrates methods to collect and translate online opinions which reside on social media platforms as well as blogs. The research evaluates different analysis approaches while presenting methods to enhance sentiment polarity detection accuracy and provides future investigation prospects for enhanced effectiveness.

Thakur et al. [22] in his previous research on Twitter discussions about online learning during COVID-19 shows that most tweets were least opinionated (56.568%), followed by neutral (30.898%) and highly opinionated (12.534%) posts, and highlights clear demographic differences in sentiment, toxicity, user activity, and country-level participation.

The Valence Aware Dictionary and sEntiment Reasoner (VADER) is an effective sentiment analysis tool optimized for social media text. Analysis of 6,232 Saudi social media posts using NLTK VADER showed 61.5% and 63.2% positive sentiment toward the first and second COVID-19 vaccine doses, respectively [23]. Another tweet based analysis with VADER produced 39.8% positive, 31.3% neutral, and 28.9% negative sentiment, while TextBlob reported 46.0% neutral, 36.7% positive, and 17.3% negative sentiment [24].

Research of Artificial Intelligence analyzed 5,000 manually labeled tweets on the HMPV virus using Word2Vec with SVM and Bi-LSTM, achieving 82.67% and 89.72% accuracy, respectively, with Bi-LSTM better capturing subtle emotional patterns [25]. Another study examined 12,340 texts from official sources, Google News, and Reddit using Naive Bayes and sentiment lexicons, finding official sources conveyed positivity, Google News emphasized negativity, and Reddit reflected fear and hope, highlighting the value of social media in health surveillance [26]. Another study on outbreak, analyzed 5,000 COVID-19 tweets using hybrid ELMo-BERT embeddings with attention, achieving 85.3% accuracy and 0.91 AUC, outperforming ELMo (82.5%, 0.87) and BERT (84.0%, 0.89) in detecting public sentiment [27]. A study on pandemics analyzed 147,475 COVID-19 tweets and 106,638 mpox tweets using Logistic Regression, Naive Bayes, RoBERTa, DistilRoBERTa, and XLNet for sentiment analysis [28]. A Twitter-based study on COVID-19 and Omicron tweets applied VADER and BERT for sentiment polarity and evaluated five supervised ML classifiers, finding predominantly negative sentiment and SVM achieving 92% accuracy with BERT on the Omicron dataset, while BERT improved performance across most models [29]. A hybrid CNN + BiLSTM with self-attention model for multi-class Amharic news classification, enhanced with XAI (LIME), achieved 97% accuracy (F1-score 96.8%) across diverse news categories, demonstrating effective and transparent text classification in a low-resource language setting [30].

The analyzed publications showcase how sentiment analysis operates across different application fields including social media inspection and customer review assessment and multimedia content emotional recognition. Future research focusing on NLP and ML improvements through analysis methods will handle existing limitations to optimize sentiment analysis applications in numerous real-world scenarios.

## 3 Methodology

In this section, the preliminaries of our methodology and the proposed approach used here is discussed. The major part of the methodology of this research is explained below:

### 3.1 Dataset

The dataset we used in our study is a collection of comments gathered from different news channels like CNN, NEWS 18, BBC, WION, Firstpost etc available on YouTube in the period of 2024–25. After data scraping, we collected 15,300 comments. After applying various preprocessing steps, the dataset was reduced to 9,758 comments. While preparing the dataset, we also labeled the comments with sentiment labels and scores. The dataset is publicly available and can be accessed at the following link [31].

#### 3.1.1 Data Preprocessing.

Data preprocessing is a critical phase for ensuring accurate evaluation and improving sentiment analysis outcomes [32]. During our preprocessing efforts, we conducted manual cross-checks to eliminate irrelevant content and reduce noise, enhancing the dataset’s reliability and integrity.

Most raw data contains noise, irrelevant information, and inconsistencies that may affect negatively impact machine learning model performance [33]. As such, effective preprocessing is very important.

**Fig 1:**
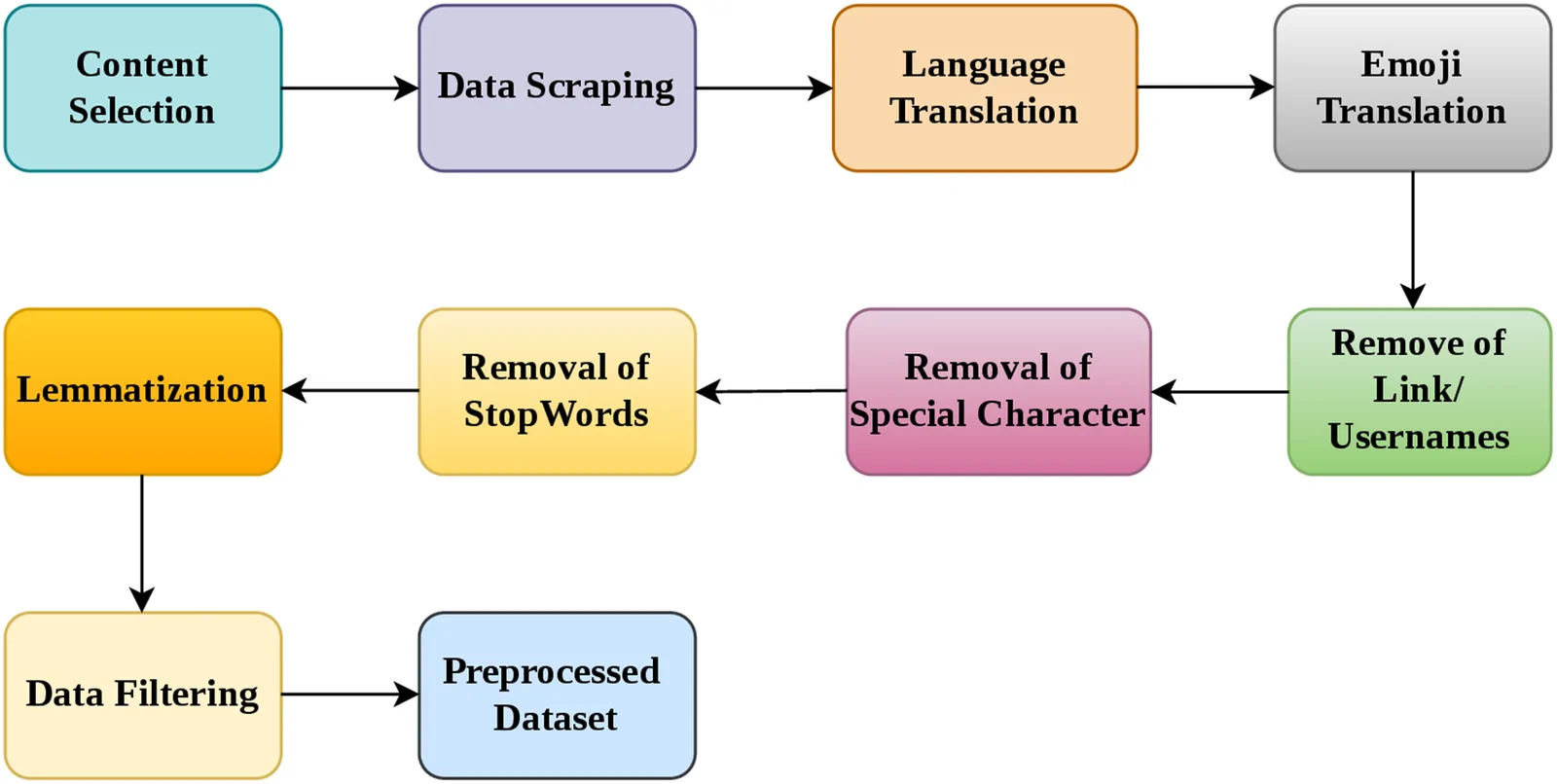
Data collection and preprocessing.

From our YouTube comment scraping, we observed significant amounts of spam, advertisements, and irrelevant discussions that provided no value for our analysis. Additionally, the dataset also included with numerous grammatical errors, typing errors, special characters, and URLs. These elements, collectively considered as noise and needed cleaning before application of machine learning models to avoid poor evaluations [34]. To address these issues, the following preprocessing steps were implemented and preprocessing steps shown in Fig 1:

**Language Translation:** Many comments were written in many languages. To apply machine learning algorithms, we had to translate all those into English. For translation we used google translator as tool.

**Emoji Translation:** Emojis tend to convey emotions that can be missed with plain text. To capture this emotion, emojis were translated into their textual equivalents to match the sentiment that they convey.

**Removal of Links, Emails, and Usernames:** Any hyperlinks, email addresses, or usernames found in the comments were removed to enhance the dataset’s focus and quality.

**Removal of Special Characters:** Special characters in the dataset were eliminated to simplify and standardize the data.

**Stopword Removal:** Common stopwords that did not contribute meaningfully to sentiment analysis were removed to reduce noise and improve model performance.

**Lemmatization:** Lemmatization was performed to convert the words into their base or root forms, maintaining the consistency and reducing dimensionality in the dataset. The task of lemmatization is to determine the basic form of a given word, which enhances the quality of text analysis [35].

By applying these preprocessing techniques, we ensured the dataset was clean, relevant and ready for analysis, significantly enhancing the overall performance of our machine learning models.

### 3.2 Data labeling

#### 3.2.1 Sentiment label identification.

For Sentiments Identification we used VADER,Which is lexion and rule based identification method [36], VADER is well suited for analyzing media texts and informal language due to its ability to capture sentiment intensity accurately. This tool classify each text such as Positive, Negative and Neutral and also provide sentiment scores which helps us for our evaluations.

### 3.3 Humanize evaluation

Simple random sampling is a commonly used evaluation approach that allows each data instance an equal or near-equal chance of being selected, thereby reducing potential sampling bias [37]. In this study, this strategy was employed to conduct a manual verification of automatically generated sentiment labels.

Initially, all comments in the dataset were labeled using VADER. It should be noted that these sentiment labels are *weak labels* generated automatically by a lexicon-based sentiment analysis method rather than a fully manually annotated gold-standard dataset. Following the automated labeling step, six subsets were randomly sampled from the dataset, each consisting of 300 comments. These sampled comments were manually reviewed by the authors to assess whether the VADER-assigned sentiment labels (positive, negative, or neutral) were appropriate in the context of the corresponding comments.

The purpose of this human evaluation was to qualitatively assess the contextual appropriateness of the automatically generated labels rather than to create a new manually annotated dataset or calculate a formal annotation agreement measure. During the manual review, the sampled comments were examined to identify any apparent inconsistencies or systematic labeling errors in the VADER-assigned sentiment labels. No systematic labeling errors were observed, and no manual relabeling of the dataset was performed. Therefore, the original VADER-generated labels were retained for the subsequent model training and evaluation. This qualitative quality check provided additional support for the suitability of the weak-labeling approach adopted in this study.

### 3.4 Model building, training and evaluation

After completing Data Collection and Data Preprocessing, We build our model and trained it. Here is an overview of our proposed model.

#### 3.4.1 XLNet model.

XLNet is an advanced natural language processing model [38]developed as an extension of the Transformer-XL architecture, introduced to overcome the limitations of previous models, such as BERT, by handling longer text sequences and leveraging permutation-based training [39]. It combines the strengths of both autoencoding and autoregressive models, capturing bidirectional context while also maintaining the autoregressive nature of language modeling. This hybrid approach allows XLNet to outperform BERT in various NLP tasks, including sentiment analysis, text classification, and question answering, by improving context understanding and sequence generation [40].

The architecture of XLNet operates with both bidirectional context care and positional encoding features through its implementation of Two-Stream Self-Attention. The pretraining and fine-tuning procedures in XLNet are comparable to BERT yet the model utilizes a novel Permutation Language Modeling (PLM) mechanism to analyze every possible input sequence permutation during pretraining. Through its distinct approach XLNet acquires additional contextual information that exceeds BERT’s capacity to perceive sequences either from left to right or right to left.

**Fig 2:**
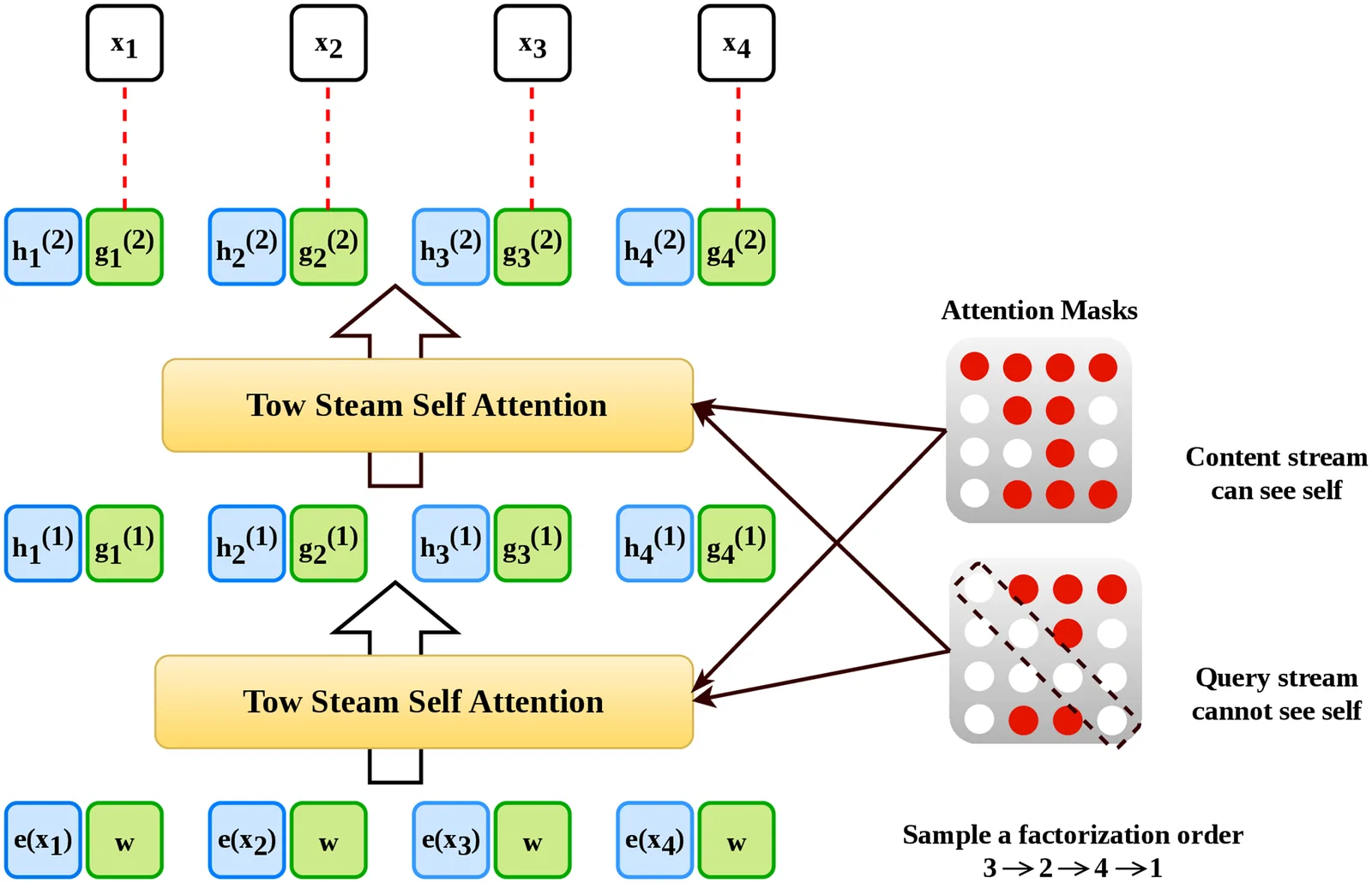
Proposed XLNet model.

**Pre-Training XLNet:** XLNet’s pre-training procedure involves Permutation Language Modeling (PLM) of extensive unlabeled text collections prior to its application on problem statements. XLNet processes sequence permutations from PLM while it learns to forecast upcoming tokens in the sequence from these permutations. Fig 2 shows the working strategy of the permutation language model(PLM). Through this method XLNet accesses every available contextual information in a sequence which enables it to obtain a flexible and enhanced understanding of text. The token relationship detection capabilities of XLNet surpass those of BERT because its permutation-based model functions without left-to-right or right-to-left direction limitations.

**Fine-Tuning XLNet:** Following pretraining the XLNet model receives specialized fine-tuning for classification of text content and answer-seeking and sentiment detection tasks. XLNet utilizes prelearned general language understanding to obtain targeted skills needed for specific tasks that demand text interpretation capabilities.

### 3.5 Hyperparameter tuning

A key performance factor of the model relies on its hyperparameter adjustment because this process directly influences both model capacity and learning speed and pattern capture abilities. Model optimization requires testing multiple parameter combinations alongside result evaluation for finding the best performance for a particular task.

The performance of the XLNet model gets affected through hyperparameter tuning since it controls how the model learns and interacts with its input data. The model depends on multiple adjustable parameters called hyperparameters such as batch size, learning rate, sequence length and number of epochs because these elements determine ultimate model success. A recently tested group of parameters presents the best outcomes in this table. Table 1 is here below.

**Table 1:** This table lists the model parameters and their corresponding values for XLNet.

Hyperparameters	XLNet Values
Learning rate (AdamW)	2e-04
Max length	50
Batch size	12
Verbose	1
Epoch	10

### 3.6 Modelevaluation

For multi-class evaluation, Precision, Recall, and F1-score were computed using weighted averaging, where each class contributes proportionally to its support [41]. This choice was made to account for class imbalance in the dataset. Accuracy, weighted Precision, weighted Recall, and weighted F1-score were computed on the same held-out test set for all transformer models to ensure fair comparison.


$$\begin{array}{c} \text{Accuracy}=\frac{\text{TP}+\text{TN}}{\text{TP}+\text{TN}+\text{FP}+\text{FN}} \end{array}$$ (1)

$$\begin{array}{c} \text{Micro F1 Score}=\frac{\sum \text{TP}}{\sum \text{TP}+\frac{1}{2}\left(\sum \text{FN}+\sum \text{FP}\right)} \end{array}$$ (2)

$$\begin{array}{c} \text{Macro F1 Score}=\frac{\sum_{i=1}^{\text{No of classes}}{\text{F1 Score}}_{i}}{\text{No of classes}} \end{array}$$ (3)

Here, TP, TN, FP, and FN indicate True Positive, True Negative, False Positive, and False Negative, respectively.

Additional equations were also used in this research to calculate Precision, Recall, Specificity, and Error Rate, as given below:


$$\begin{array}{c} \text{Precision}=\frac{\text{TP}}{\text{TP}+\text{FP}}\times 100\% \end{array}$$ (4)

$$\begin{array}{c} \text{Sensitivity/Recall}=\frac{\text{TP}}{\text{TP}+\text{FN}}\times 100\% \end{array}$$ (5)

$$\begin{array}{c} \text{Specificity}=\frac{\text{TN}}{\text{TN}+\text{FP}} \end{array}$$ (6)

$$\begin{array}{c} \text{Error Rate}=\frac{\text{FP}+\text{FN}}{\text{TP}+\text{TN}+\text{FP}+\text{FN}} \end{array}$$ (7)

### 3.7 Model explainability

To improve transparency and interpretability of the transformer-based sentiment classifier, SHAP (SHapley Additive exPlanations) was employed to analyze feature contributions in the XLNet model. Given the complexity and non-linearity of transformer architectures, careful design choices were made to ensure meaningful and stable explanations [42,43].

**Explainer Type:** We utilized the **SHAP PartitionExplainer**, which is suitable for deep neural networks and more computationally efficient and stable than KernelExplainer for high-dimensional text inputs. The explainer was applied to the fine-tuned XLNet model in inference mode.

**Background Dataset Selection:** The background (reference) dataset used by SHAP consisted of a randomly sampled subset of **100 neutral comments** from the training data after preprocessing. This choice provides a representative baseline distribution while avoiding bias toward extreme sentiment classes. Using a neutral background helps stabilize Shapley value estimation for sentiment attribution.

**Tokenization and Subword Handling:** XLNet employs subword tokenization based on SentencePiece. SHAP attributions were initially computed at the **subword-token level**. To improve interpretability, subword attributions corresponding to the same original word were **aggregated by summation** to produce word-level importance scores. This aggregation enables clearer visualization and interpretation of sentiment-driving words in user comments.

**Interpretation and Stability:** SHAP values indicate the contribution of each word toward pushing the model prediction toward a specific sentiment class (positive, neutral, or negative). Positive SHAP values increase the likelihood of the predicted class, while negative values decrease it. SHAP explanations were generated only for correctly classified samples to facilitate the interpretation of the model’s learned decision patterns. Misclassified instances may reflect prediction errors, ambiguous language, or limitations of the training data, making it more difficult to distinguish meaningful feature contributions from error-specific behavior. Therefore, correctly classified samples were selected to illustrate representative explanations of the model’s decision-making process rather than to evaluate its behavior on erroneous predictions.

By explicitly defining the explainer configuration, background data, and token aggregation strategy, the SHAP analysis provides reliable and interpretable insights into the decision-making process of the XLNet-based sentiment classifier.

**Fig 3:**
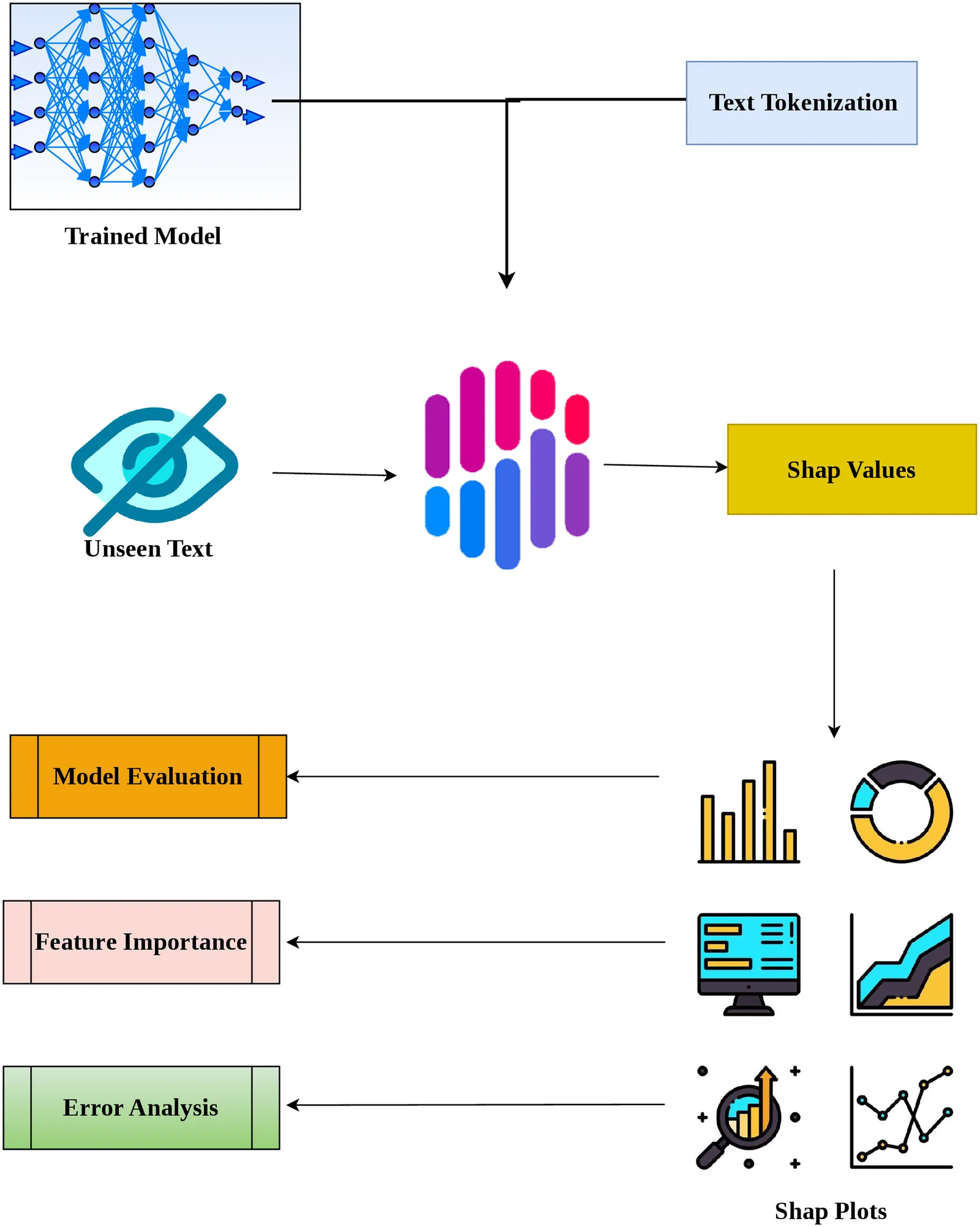
The working process of SHAP.

Fig 3 illustrates the working principle of the SHAP algorithm, showing how individual features contribute to sentiment classification for comments on the HMPV virus.

### 3.8 Proposed approach

**Fig 4:**
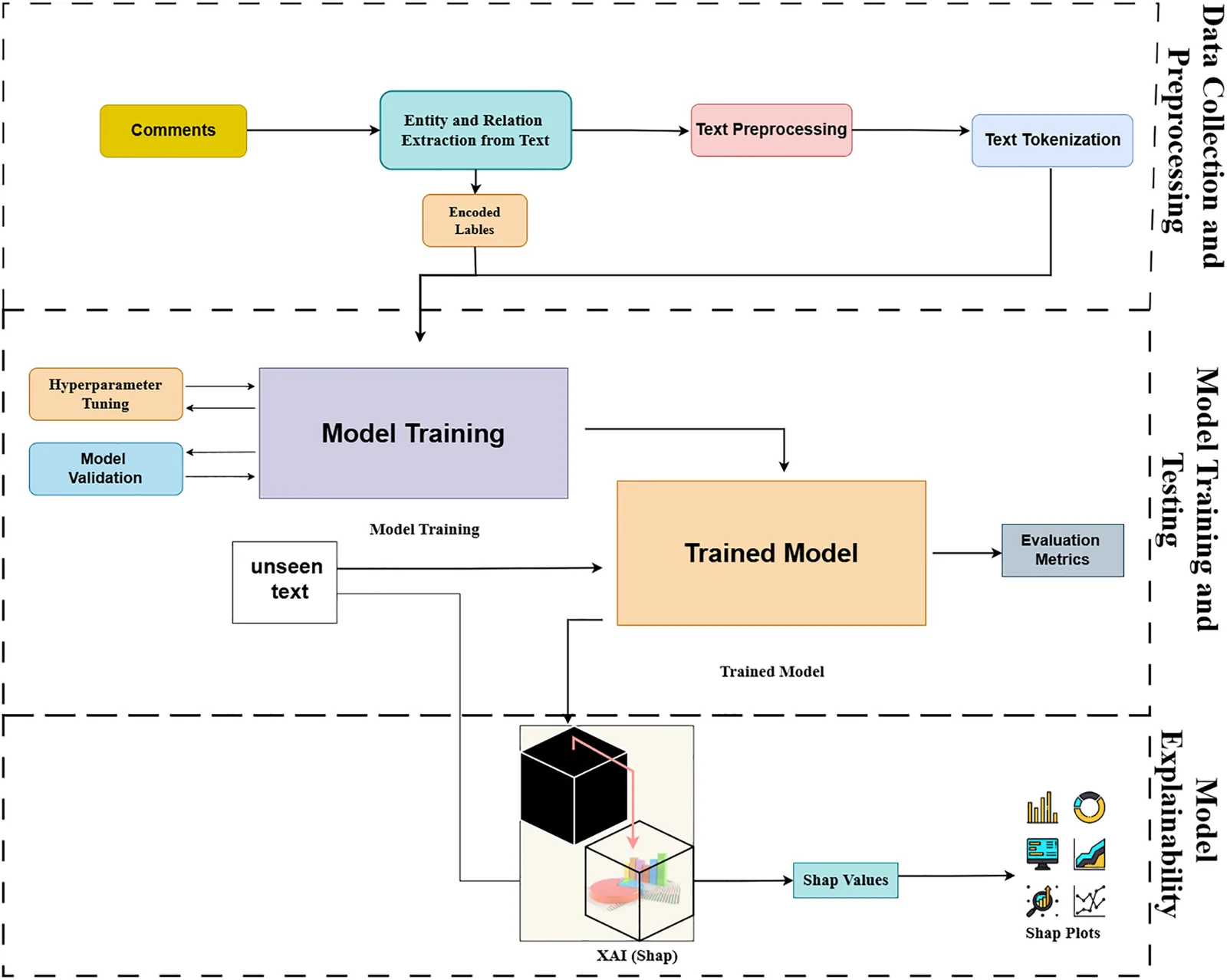
Workflow of the proposed approach.

Fig 4 delineates the workflow diagram of the approach proposed for sentiment analysis of comments related to the HMPV virus. The entire workflow can be divided into three distinct parts.

In the first division, a thorough and step-by-step data collection and preprocessing process is outlined. The process begins with extracting essential features from user comments followed by processing and tokenizing their text before assigning sentiment labels which can be either positive or negative or neutral. Online platform users typically submit comments that go through cleaning operations to create high-quality inputs for the model’s training process.

Next, the second division focuses on model building and training. This includes essential steps like hyperparameter tuning, model selection, and evaluation using metrics like accuracy, micro and macro F1 scores. After these steps, a trained model is obtained that can predict the sentiment of previously unseen comments related to the HMPV virus.

Finally, the Explainability division applies the SHAP algorithm to the trained model using unseen data. SHAP values are computed to provide insights into the decision-making process of the model. By using the SHAP algorithm and the model’s tokenizer, the SHAP values are determined for each unseen comment, allowing us to understand how specific words or phrases in the comments influence the model’s sentiment predictions.

### 3.9 Ethical considerations and data privacy

This study analyzed publicly available user comments collected from YouTube news channels. All data were accessed in accordance with YouTube’s Terms of Service and did not involve any interaction with users, private accounts, or restricted content. No attempts were made to bypass platform safeguards or access non-public information.

To protect user privacy, all personally identifiable information (PII), including usernames, profile references, URLs, email addresses, and external links, was removed during the preprocessing stage. The dataset contains only anonymized textual content and does not include metadata that could be used to identify individual users.

As the data consist solely of publicly accessible comments and were analyzed in aggregate for research purposes, the study does not involve human subjects as defined by institutional review standards and therefore did not require formal ethics committee approval. Nevertheless, ethical best practices for social media research were followed, including data minimization, anonymization, and responsible reporting. The findings are presented at the population-discourse level, and no individual comments are attributed to identifiable users.

## 4 Results

A comprehensive evaluation was conducted by applying various transformer-based architectures, including ELECTRA, RoBERTa, ALBERT, DistilBERT, XLNet, and BERT, to the preprocessed dataset. Each model was trained using its corresponding tokenizer, and performance was assessed using four key evaluation metrics: Accuracy, Precision, Recall, and F1 Score. The summarized results, presented in Table 2, highlight that XLNet achieved the best performance across the metrics. While weighted metrics are reported in Table 2 for overall comparison, class-wise and macro-average results are presented in Table 3 to provide a more granular evaluation.

**Table 2:** Performance comparison of transformer-based models using weighted-average Precision, Recall, and F1-score on the same test set.

Model	Accuracy (%)	Precision (%)	Recall (%)	F1 Score (%)
RoBERTa	89.96	90.12	89.96	89.97
ELECTRA	91.14	91.37	91.14	91.15
ALBERT	89.60	89.67	89.60	89.57
DistilBERT	89.50	89.46	89.50	89.45
XLNet	93.50	92.50	93.50	93.00
BERT	91.00	91.00	91.00	91.00

**Table 3:** Classification report showing precision, recall, and F1 score for each class.

Class	Precision (%)	Recall (%)	F1 Score (%)	Support
Negative	91.00	96.00	93.00	743
Neutral	99.00	92.00	95.00	712
Positive	88.00	90.00	89.00	497
**Accuracy**		93.5		1952
**Macro Avg**	93.0	93.0	93.0	1952
**Weighted Avg**	93.0	93.0	93.0	1952

**Fig 5:**
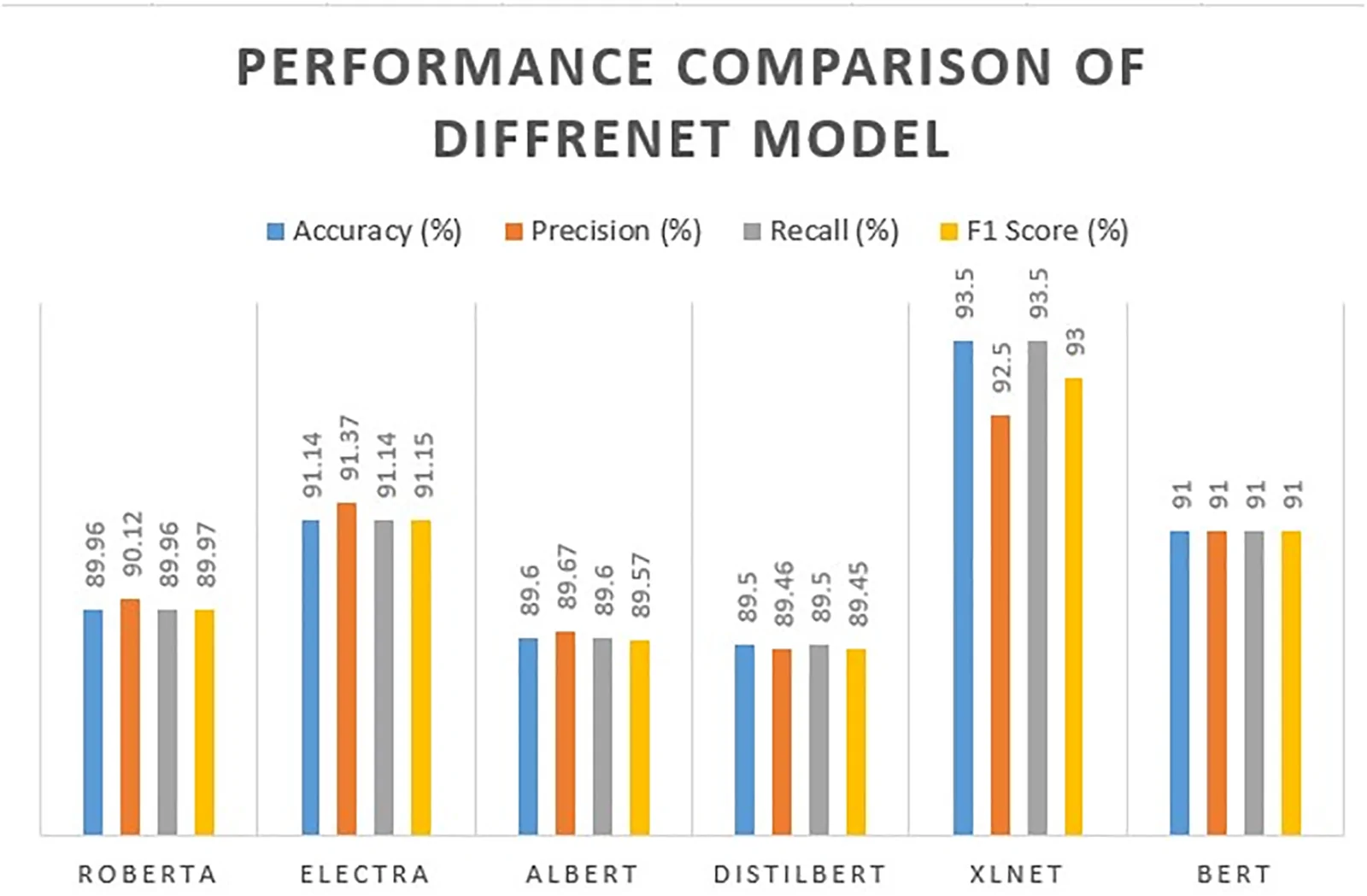
Comparison of accuracy, precision, recall, and F1 Score for different transformer models: BERT, ELECTRA, RoBERTa, ALBERT, DistilBERT, and XLNet.

Among the evaluated models, XLNet outperformed the others with an accuracy of **93.50%**, followed by ELECTRA (**91.14%**) and BERT (**91.00%**). RoBERTa, ALBERT, and DistilBERT demonstrated lower accuracy scores, ranging from **89.50%** to **89.96%**. Fig 5 visually illustrates the comparative performance of these models, with XLNet showing consistent advantages in all evaluation metrics.

### 4.1 Confusion matrix analysis

**Fig 6:**
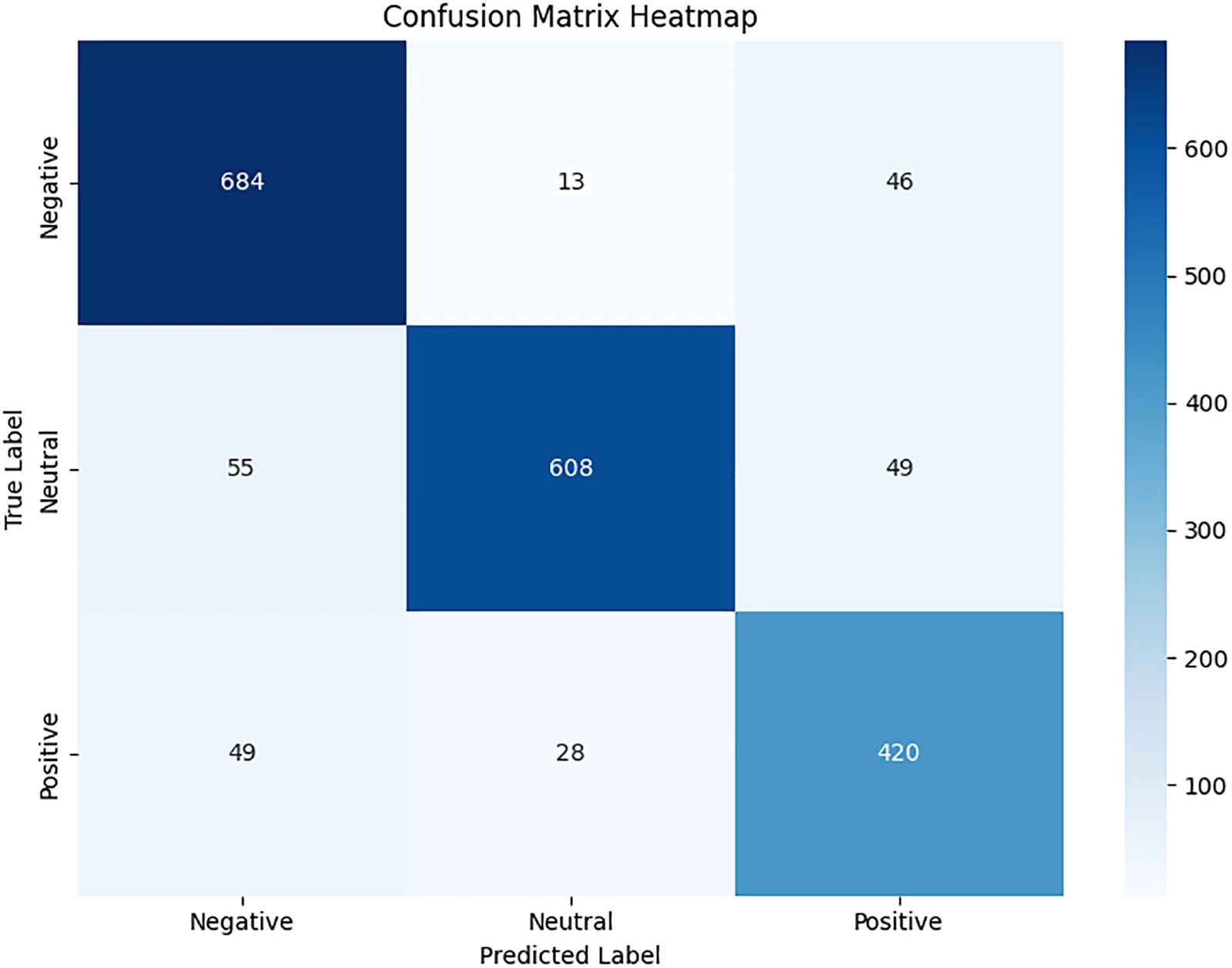
Confusion Matrix of the classification model. The diagonal values represent correct predictions, while the off-diagonal values indicate misclassifications.

To further analyze model performance, a confusion matrix was generated, as shown in Fig 6. The diagonal elements of the matrix represent correct classifications, while off-diagonal elements indicate misclassifications. The model correctly classified **708 instances** of the Negative class, **629 instances** of the Neutral class, and **424 instances** of the Positive class. However, some misclassifications were observed:

- **Negative Class**: 12 instances misclassified as Neutral, 23 instances misclassified as Positive.
- **Neutral Class**: 48 instances misclassified as Negative, 35 instances misclassified as Positive.
- **Positive Class**: 56 instances misclassified as Negative, 17 instances misclassified as Neutral.

These results indicate that the model performs well in distinguishing the Negative and Neutral classes, while the Positive class exhibits a moderate misclassification rate. This suggests that additional fine-tuning, data augmentation, or feature engineering may be required to improve the model’s ability to differentiate the Positive class effectively.

### 4.2 Detailed classification report

The confusion matrix was further analyzed to compute additional evaluation metrics, including Precision, Recall, Specificity, and Error Rate. Table 3 presents the detailed classification report for each class, including Micro and Macro Averages.

### 4.3 ROC curve analysis

To evaluate the discriminatory capability of the proposed sentiment classification model, Receiver Operating Characteristic (ROC) analysis was performed. Since the task involves multiclass sentiment classification, a **one-vs-rest (OvR)** strategy was adopted, where each sentiment class (Negative, Neutral, and Positive) was evaluated against the remaining classes.

For each class, ROC curves and corresponding Area Under the Curve (AUC) values were computed using the **softmax probability outputs** of the trained XLNet model. No additional probability calibration techniques (e.g., Platt scaling or temperature scaling) were applied. All ROC and AUC computations were conducted on the same held-out test set to ensure consistent and fair evaluation across classes.

**Fig 7:**
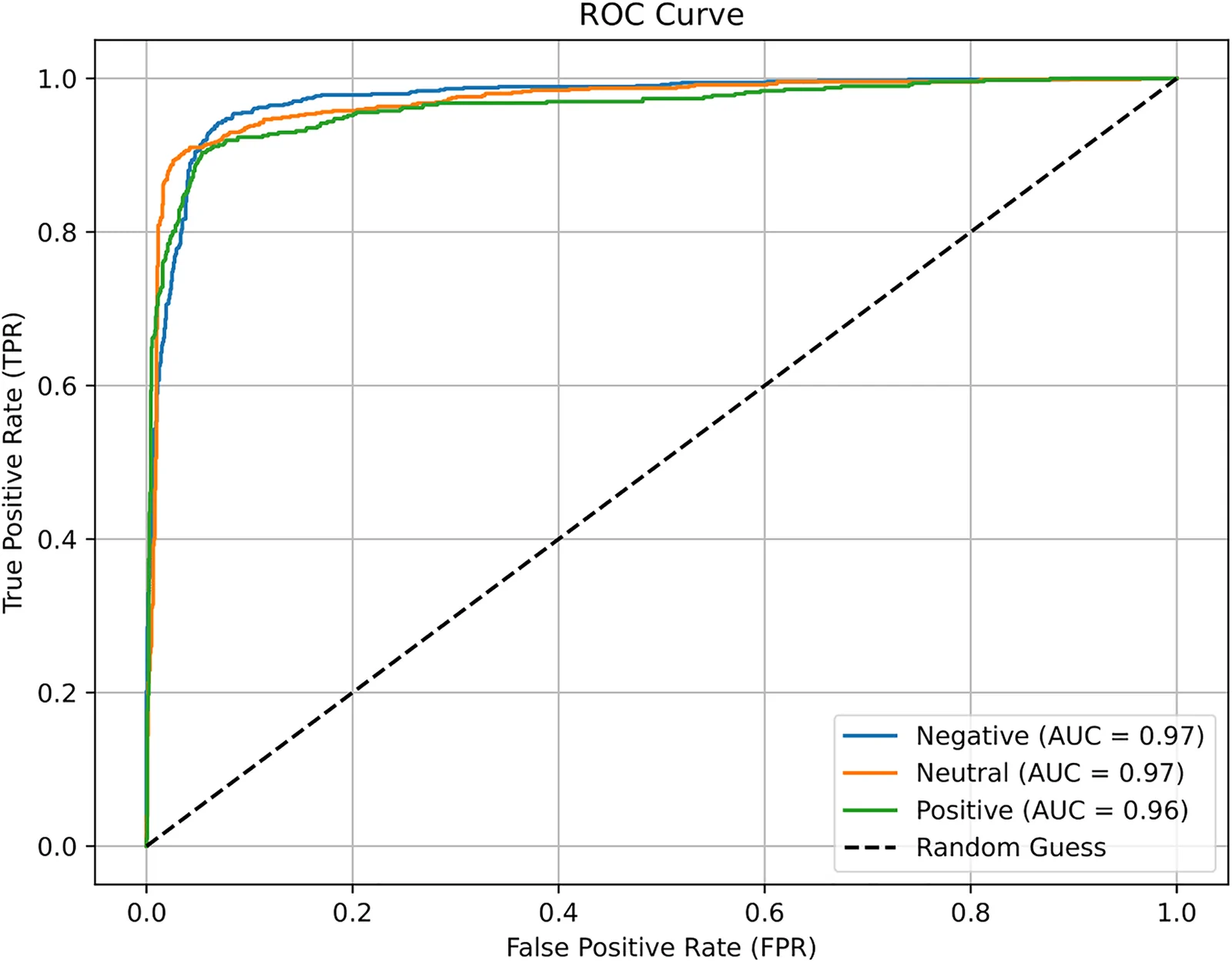
One-vs-rest (OvR) ROC curves for each sentiment class using softmax probability outputs of the XLNet model.

Fig 7 presents the OvR ROC curves for each sentiment category. The Negative and Neutral classes achieved AUC values of **0.97**, while the Positive class obtained a slightly lower AUC of **0.96**. These high AUC values indicate the model’s strong ability to distinguish each sentiment class from the others. Classwise AUCs are reported to provide interpretable, sentiment-specific performance insights, rather than relying solely on aggregated metrics.

### 4.4 SHAP analysis for model interpretability

**Fig 8:**
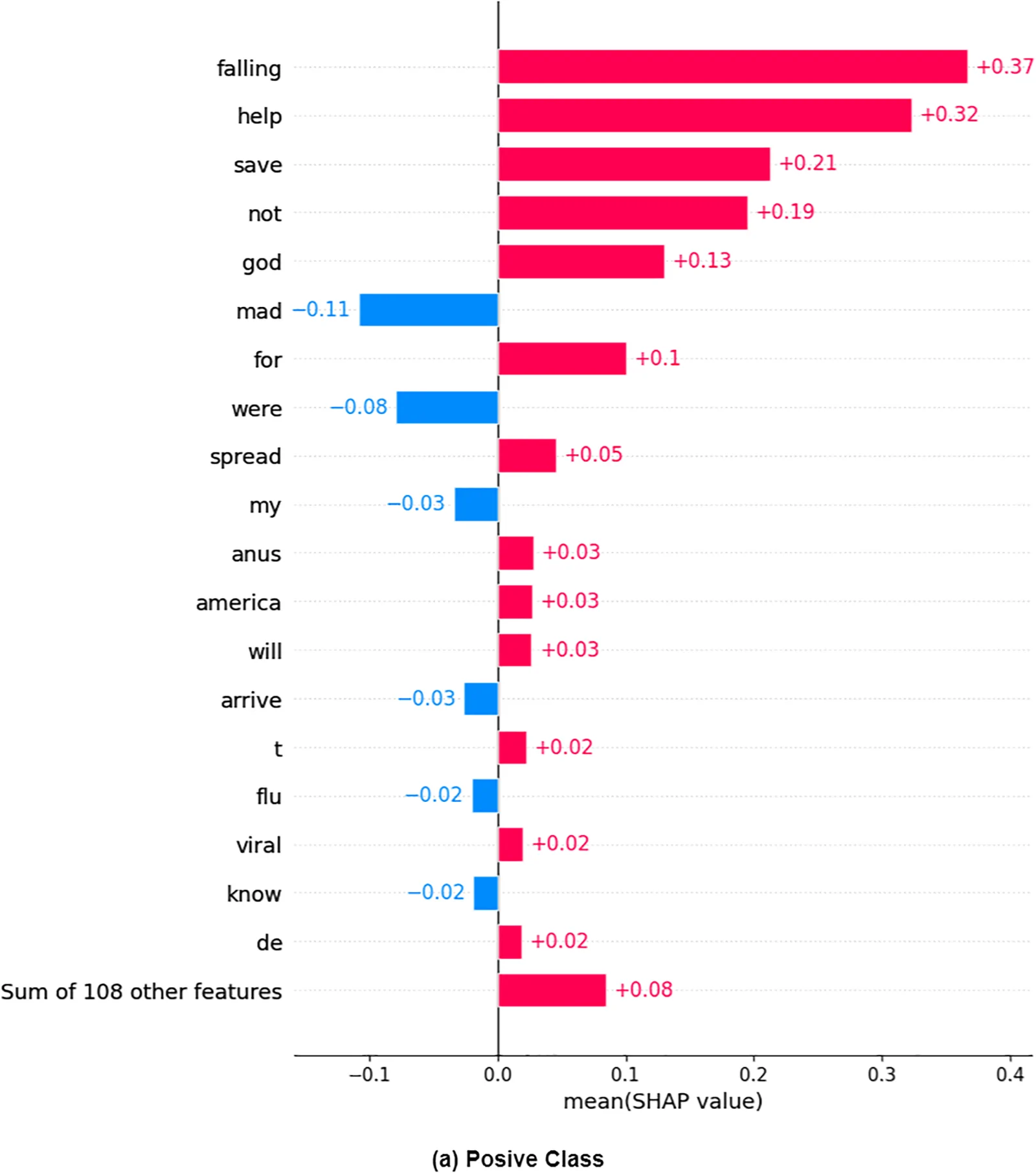
SHAP summary plot illustrating the key features influencing model predictions.

**Fig 9:**
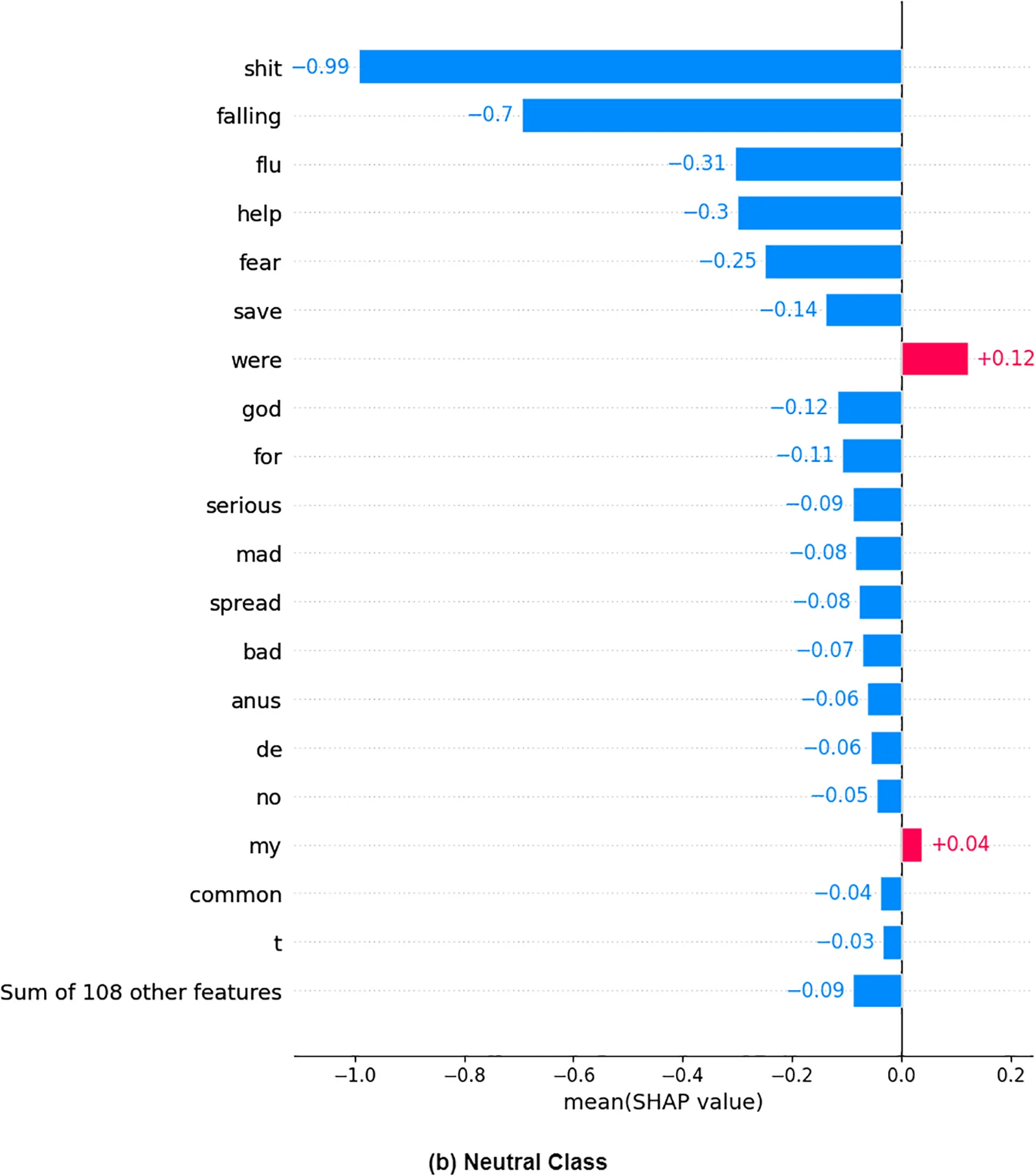
Word-level SHAP explanations for XLNet predictions. Subword-level attributions were aggregated to words using summation. Red features contribute toward the predicted sentiment, while blue features contribute against it.

**Fig 10:**
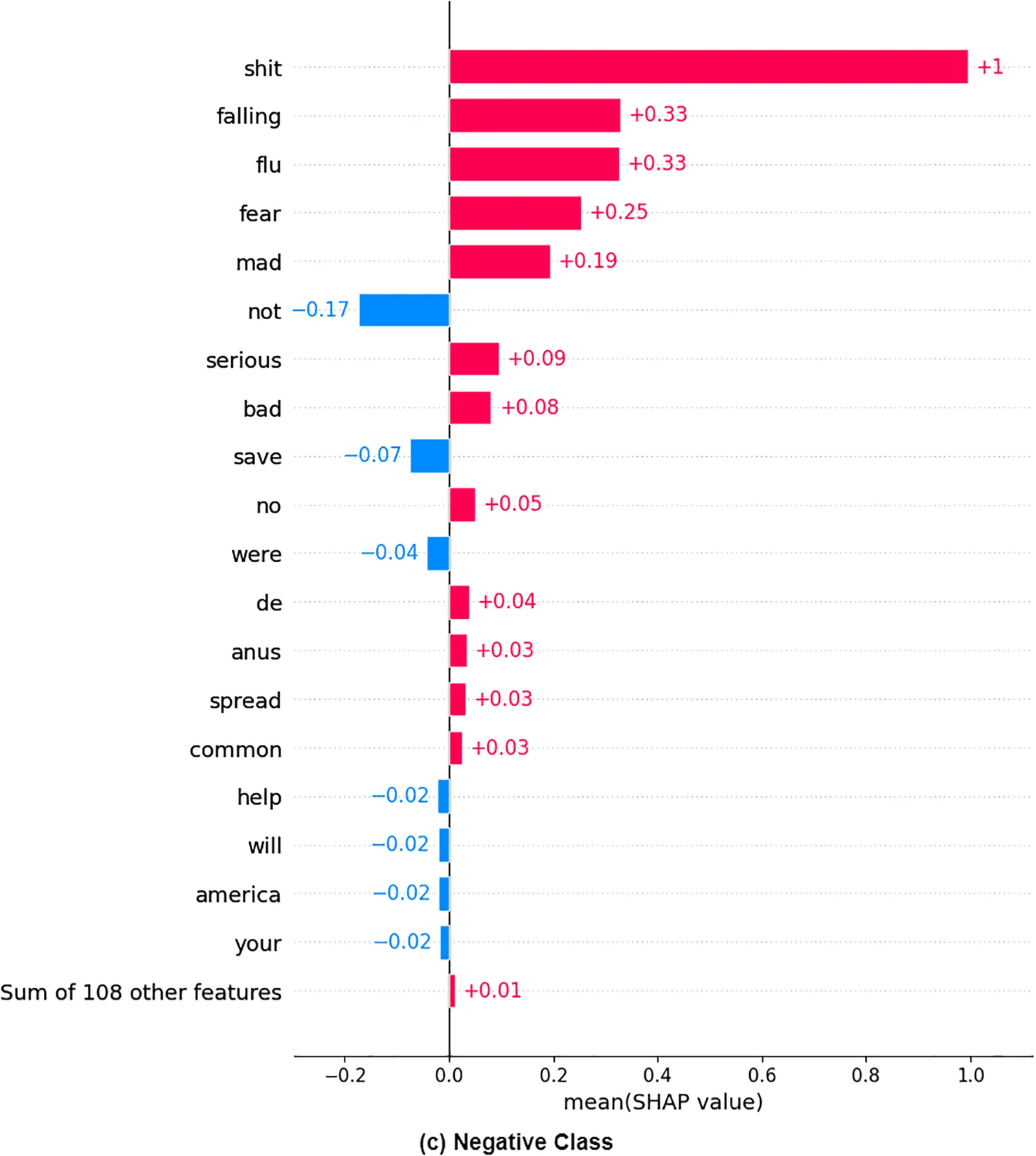
SHAP analysis showing the impact of individual words on model predictions. Words contributing to negative sentiment are highlighted in red, while those contributing to neutrality or positivity are marked in blue.

To further validate XLNet as the optimal model, the SHAP (SHapley Additive exPlanations) explainer was utilized. SHAP values provide interpretability by identifying important features influencing predictions. Figs 9 and 10 present SHAP force plots, which illustrate how individual words contribute to sentiment classification. Fig 8 present SHAP summary plots.

The SHAP analysis reveals that specific words significantly impact the model’s predictions. The force plots in the figures show how each word pushes the classification toward either a positive, neutral, or negative sentiment. For example:

- Words such as *“flu,” “fear,”* and *“shit”* have high positive SHAP values in negative sentiment predictions, highlighting their strong contribution to negative classification.
- Words like *“save,” “help,”* and *“mad”* exhibit high SHAP values in neutral sentiment predictions, demonstrating their influence in maintaining neutrality.
- Positive sentiment classifications are driven by words such as *“falling,” “save,” “god,”* and *“america,”* which push the sentiment away from negativity.

The force plots also demonstrate the cumulative effect of multiple words in determining the final classification. Words with strong SHAP values in either direction directly influence whether the sentiment is classified as negative, neutral, or positive.

The experimental results indicate that XLNet achieved the best performance among the transformer-based models evaluated in this study for sentiment classification of HMPV-related YouTube comments. The SHAP analysis provides interpretability by identifying key linguistic features influencing predictions. The confusion matrix, classification report, and ROC analysis further validate the model’s robustness and accuracy, confirming its effectiveness for sentiment analysis tasks.

### 4.5 Qualitative validation of SHAP-identified important words

While SHAP provides approximate feature-attribution scores, interpretation of “important words” in transformer-based models requires qualitative validation to avoid misleading conclusions arising from topic drift, spam, or labeling artefacts. To address this concern, we conducted a targeted human-centered qualitative analysis of SHAP-identified terms. For each sentiment class (Negative, Neutral, and Positive), we randomly sampled **50 comments** from the correctly classified test instances in which SHAP assigned high absolute importance values to specific words. These sampled comments were manually reviewed by the authors to assess whether the highlighted words were contextually related to HMPV discourse or reflected unrelated topics such as political discussion, general profanity, or spam. The qualitative review revealed that the majority of high-importance words occurred within comments explicitly discussing HMPV-related concerns, including infection severity, public health response, personal anxiety, or trust in authorities. For example, words such as *“flu”*, *“fear”*, and profane terms (e.g., *“shit”*) were commonly embedded in emotionally charged expressions describing perceived health risks or frustration with outbreak management, rather than appearing as isolated or spam-related tokens. In cases where seemingly generic or ambiguous terms (e.g., *“america”*, *“falling”*, *“mad”*) were assigned high SHAP importance, contextual inspection showed that these words were typically used in relation to news reporting, international spread of HMPV, or public reactions to governmental health messaging. A small proportion of comments (< 10%) were identified as partially off-topic or weakly related to HMPV; however, these instances did not dominate the SHAP importance distributions. This qualitative validation indicates that SHAP-identified “important words” largely align with meaningful HMPV-related discourse rather than reflecting topic drift, spam, or systematic mislabeling. Nevertheless, we emphasize that SHAP explanations represent approximate contribution estimates rather than causal attributions, and should be interpreted in conjunction with contextual analysis.

## 5 Discussion

This research created a reliable sentiment analysis system which classifies HMPV virus-related YouTube comments through developing techniques for data preprocessing along with model development and explainability phases. The investigation shows how handling multilingual noisy data presents difficulties which XLNet and SHAP successfully resolve.

Machine learning required thorough data preprocessing preparation to utilize the dataset. A large fraction of the initial comments included messages which were not relevant to the study thus the preprocessing pipeline proved essential for improving the reliability of the dataset. Multiple operations including language translation, emoji translation, link and special character removal, stopword elimination and lemmatization were mandatory to transform comments into an analyzable and uniform format. The data-preparation steps succeeded in cutting down unnecessary noise due to their importance in producing accurate model predictions. The manual cross-checks conducted further contributed to the integrity of the data, as they ensured that only the most relevant content was retained, as shown in 1.

The XLNet model proved superior to traditional models like BERT because it excels at processing long series and understanding both directions of context for sentiment analysis operations. Permutation-based training enables XLNet to identify token relations better which makes it suitable for processing informal user-generated content available on platforms such as YouTube comments effectively. The model acquired ability to detect online comment sentiment through pretraining followed by fine-tuning that enabled its adaptation to the special behavior found in sentiment detection tasks. These processes demonstrated how powerful NLP models work with NLP tasks in research presented in 5.

Hyperparameter tuning also played a significant role in optimizing the XLNet model. By adjusting key parameters such as learning rate, batch size, and number of epochs, we were able to improve the model’s convergence speed and overall performance. The optimal hyperparameters, identified through experimentation, allowed the model to strike a balance between overfitting and underfitting, ensuring reliable predictions on unseen data. 1 presents the final set of tuned hyperparameters.

The model’s evaluation metrics—accuracy, micro and macro F1 scores, precision, recall, specificity, and error rate—demonstrated the effectiveness of the model in classifying sentiment accurately. The use of multiple evaluation metrics provided a comprehensive view of the model’s performance, ensuring it was not biased toward a particular class. These results emphasize the importance of considering various metrics when assessing a model, especially in real-world applications where data can be imbalanced, as shown in 2.

The research incorporated SHAP for model explainability as its primary important advancement. SHAP analysis provided essential transparency to the XLNet model through identification of the comment features which impacted predicted sentiments. The interpretability of the model is essential for user trust because it helps users follow the model decisions while identifying possible enhancement points. Through SHAP insights we could perform error analysis which identified the particular comment features responsible for incorrect predictions. This feedback loop enables continuous model refinement, which is essential for real-world deployment, as shown in 8.

Despite the promising results, there are several limitations to the current study. While XLNet demonstrated strong performance, it requires significant computational resources, especially during pretraining, which can be a barrier for smaller-scale applications. Additionally, the reliance on sentiment labeling may not always capture the full complexity of user emotions, as some comments may contain mixed sentiments or sarcasm, which are challenging to identify. Future research could explore integrating multimodal data, such as video or image content, along with text, to enhance sentiment detection in multimedia platforms like YouTube.

### 5.1 Limitations and scope of public health inference

While the proposed framework provides valuable insights into public sentiment surrounding HMPV, several limitations should be acknowledged. First, the analysis is based exclusively on YouTube comments collected from selected international news channels. As such, the findings reflect **platform-specific discourse** rather than population-level public opinion. Social media users are subject to demographic, geographic, and self-selection biases, and YouTube commenters may not be representative of the broader public. Additionally, platform-specific dynamics—such as algorithmic amplification, moderation policies, and engagement-driven commenting behavior—can influence sentiment expression. Consequently, the observed sentiment patterns should not be interpreted as direct indicators of public attitudes or behavioral intentions at the population level. Therefore, the public health relevance of this study lies primarily in its ability to support **exploratory infodemiological analysis** and early detection of online discourse trends, rather than in making direct policy or intervention recommendations. Future work may incorporate multi-platform data (e.g., Twitter/X, Facebook, Reddit), demographic metadata where ethically permissible, and triangulation with survey-based or epidemiological data to improve representativeness and external validity.

## 6 Conclusion and future work

A sentiment analysis system was created to identify HMPV-related YouTube user commentary while resolving issues affecting unstructured multilingual noisy data types. Advancements in NLP techniques allowed the utilization of XLNet and SHAP to reveal useful insights from user-generated content. Preprocessing steps of language translation combined with emoji translation and stopword removal played an essential part in enhancing both data quality and model performance.

The sentiment classification method we introduce employing XLNet surpasses traditional models resulting in successful performance and impressive scores for accuracy and complementary metrics. The implementation of SHAP enabled better model explainability which produced valuable information about feature significance as well as maintaining the capability to identify errors. The combination of XLNet and SHAP demonstrates strong performance and provides interpretable predictions for sentiment analysis of HMPV-related YouTube comments, suggesting its potential usefulness for similar social media analysis tasks.

Several aspects need progress even though the model demonstrates some improvements. XLNet and similar huge models present a training cost challenge which poses difficulties for environments with limited resources. Future robustness of the model can be improved by resolving technological issues with combined sentiments and sarcasm detection and handling multiple data types. Future research should investigate better techniques to manage noisy and adversarial data because this would improve model effectiveness across different conditions.

The main objective of upcoming work involves improving the XLNet model for use in live-time systems. The project extends the current YouTube comment dataset for improvement of the model’s generalization capabilities and operational strength. Our future work includes the assessment of incorporating user metadata and video content to accomplish a deeper understanding of user sentiment. The development of fast lightweight adaptations for the model represents a vital necessity for achieving real-time accuracy performance without trading off precision standards.

This investigation contributes to sentiment analysis research on social media by demonstrating the application of advanced transformer-based NLP models and interpretable AI techniques for comment classification. Our work provides a foundation for future research on sentiment analysis systems that combine strong predictive performance with interpretable decision-making.
